# FPFS-YOLO: An Insulator Defect Detection Model Integrating FasterNet and an Attention Mechanism

**DOI:** 10.3390/s25134165

**Published:** 2025-07-04

**Authors:** Yujiao Chai, Xiaomin Yao, Manlong Chen, Sirui Shan

**Affiliations:** School of Mechanical Engineering, Shaanxi University of Technology, Hanzhong 723000, China; 15667263939@163.com (Y.C.); hz202053@126.com (M.C.); shansirui0726@163.com (S.S.)

**Keywords:** defect detection, minor-defect detection layer, FasterNet network structure, PConv, YOLO

## Abstract

The timely detection of insulator defects in transmission lines is vital for ensuring social production and people’s livelihoods. Aiming to solve the problem of the low accuracy of insulator defect detection in current detection models, this study improves the YOLO11n model and proposes an insulator defect detection model, FPFS-YOLO, that integrates FasterNet and an attention mechanism. In this study, to mitigate parameter redundancy in the backbone of the YOLO11n model, the FasterNet lightweight network was introduced, and some convolution was embedded into the shallow network to enhance its feature extraction ability. To solve problems such as insufficient attention to important features and the low detection ability of small defects in the YOLO11n model network, the ParNet attention mechanism was added, along with a small-defect detection layer, which improved the detection accuracy of the model. Finally, in order to alleviate the computational redundancy caused by these additions, the C3k2_faster module and the PSP-Head detection head were introduced. These amendments further improved the accuracy of the model network in detecting insulator defects while simultaneously reducing its computational redundancy. The experimental results show that the improved FPFS-YOLO model achieved a 91.5% mAP@50 and a 56.6% mAP@0.5-0.95, increases of 3.1% and 1.2%, respectively, while the precision and recall reached 93.2% and 86.4%, increases of 1.5% and 4.2%, respectively. The FPFS-YOLO model achieved a higher detection accuracy than the YOLO11n model and thus could be widely applied in the detection of insulator defects.

## 1. Introduction

Transmission lines are usually found in complex and changing outdoor environments, and insulators, as their key components, endure long-term exposure to the outdoors, not only facing the challenges of this external environment but also withstanding power loads and other internal pressures. Therefore, ensuring their safe and stable operation is vital in safeguarding social production and people’s lives [1,2]. With the increasing development and application of technologies such as drones and artificial intelligence, traditional manual inspection methods are no longer sufficient for efficient and accurate modern power system monitoring. Therefore, in order to improve inspection efficiency, reduce safety risks, and provide a strong guarantee of the stable operation of the power system, combining drone and deep learning research is an inevitable trend in the development of the power grid’s intelligence. Drones fly according to predetermined trajectories [1] and take corresponding videos or images. Then, with the help of image detection technology, inspections of transmission lines can be carried out. The core task in drone inspections is to quickly and accurately identify insulator defects to provide more accurate data support for subsequent maintenance work [3,4].

Osco et al. [5] analyzed 232 studies in the international scientific journal databases to analyze the role of deep learning networks in the field of remote sensing and the role of unmanned aerial vehicles in the direction of aerial remote sensing. This paper focuses on elaborating the application of classification and regression techniques in scenarios such as environmental monitoring, urban planning, and agricultural management. By analyzing sensor types, technical architectures, and application characteristics, it reveals the potential of deep learning in processing unmanned aerial vehicle (UAV) data and looks forward to future frontier directions such as real-time processing and domain adaptive learning.

Carrio et al. [6] discussed the application of unmanned aerial vehicles (UAV) based on deep learning. Since CNN has a strong ability to extract images, it was concluded that the deep learning algorithms it uses in UAV applications are dominant. In the paper, the challenges currently faced were also analyzed, such as the autonomous operation of unmanned aerial vehicles (UAV), which require a large amount of computing resources, and how to develop embedded hardware and design more efficient deep learning architecture.

GERALDES et al. [7] designed and developed a novel situation awareness system based on unmanned aerial vehicles (UAV), Person-Action-Locator (PAL), which can be used for tasks such as search, surveillance and rescue, and is embedded into the NVIDIA Jetson Tegra X2. However, due to the problem of computing resources, it is difficult to obtain real-time detection. In the field of deep learning, target detection algorithms are categorized according to the flow and complexity of the processing task [8,9] into two main categories, namely two-stage [10] and one-stage target detection algorithms [11,12]. Two-stage target detection algorithms exhibit more detailed and step-by-step processing flow in the detection process, as seen for R-CNN [13], Faster R-CNN [14], etc. Single-stage target detection algorithms such as SSD (Single-Shot Multi-Box Detector) [15] and YOLO (You Only Look Once) [16,17], on the other hand, accomplish detection tasks quickly and efficiently. Compared with two-stage target detection algorithms [18], single-stage target detection algorithms, represented by the YOLO series, have been applied more widely in the field of target detection by virtue of its advantage in balancing speed and accuracy, especially in application scenarios with high real-time requirements, such as UAV inspections.

In recent years, the use of UAVs to detect insulator defects has become a hot research topic. Li et al. [19] proposed an FPEF-SSD target detection algorithm based on a feature-enhanced fusion SSD with a pyramid network in order to improve its accuracy for small-target detection. The accuracy of this algorithm was improved by 8.2%, but its ability to distinguish similar small targets still needed to be improved. Shen et al. [20] improved adaptive spatial feature fusion by passing the features through multi-scale feature fusion based on the YOLOv5s model and used the SIoU loss function to accelerate convergence and improve the accuracy of the model. Their mAP@50 value improved by 5.1%, but missed and false detections also occurred. Wei et al. [21] addressed the problems of insufficient data and low accuracy by adding CBAM to the neck of the YOLOv5s model and transforming the dimensionality of the input feature maps, which improved its ability to extract and fuse the defect features of small targets and improved the mAP by 3.02%. Cao et al. [22] introduced feature-reuse-based CSO and CAM into the feature extraction layer of the YOLOv8m model to create the CACS-YOLO model, which showed an improved detection accuracy. Their model reached a 96.9% mAP@0.5-0.95 value, but its Params value was 27.44 M and its FLOPS value was 66.6 G.

In summary, existing studies have shown the need to improve detection accuracy while keeping computational effort low. Moreover, due to the introduction of a refined architecture design and an optimized training process by YOLO11n, it achieves a faster processing speed while maintaining the best balance between accuracy and performance. In this study, the YOLO11n model’s network structure is optimized and the FPFS-YOLO (FasterNet-ParNet-C3k2_faster-SE) model is proposed. Its key innovations are as follows:

(1) To address the low detection accuracy of the YOLO11n model, this study introduces the FasterNet lightweight network, PConv, the ParNet attention mechanism, and a small-defect detection layer into its network structure. Through this multi-level network, its ability to detect, extract, and fuse the features of small defects is enhanced.
(2) To tackle the computational redundancy caused by the introduction of the ParNet attention mechanism and the small-defect detection layer into the model, this study introduces the C3k2_faster module and designs the PSP-Head detection head, which further improve the detection accuracy while reducing the computational volume of the model and its number of parameters.

## 2. Methods

### 2.1. YOLO11n Introduction

**Figure 1:**
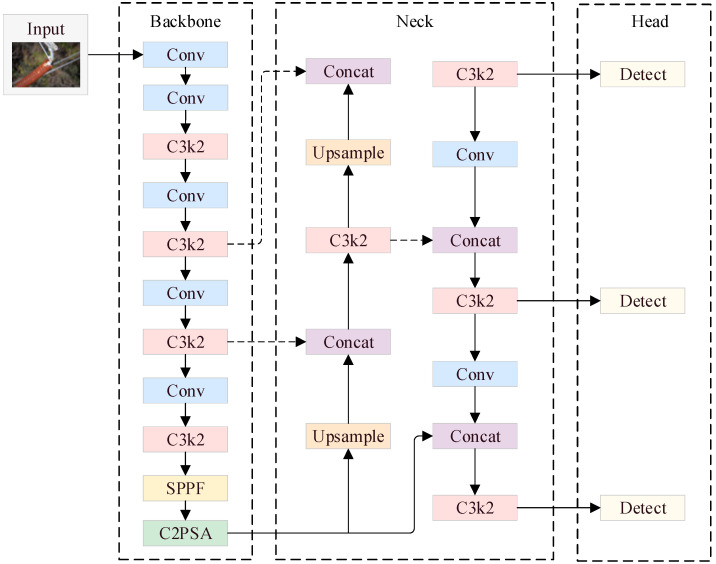
YOLO11n network structure.

YOLO11 [16] is a target detection algorithm that was released by the Ultralytics team in 2024. It is capable of carrying out various computer vision tasks using its powerful and rich functions. The YOLO11 network structure is shown in Figure 1, consisting of the backbone, the neck, and the head.

Compared with the previous YOLO series, the main innovations of the YOLO11 model include an optimized backbone network, an improved feature fusion mechanism, an efficient detection head design, and enhancements in small object detection. First, the original C2f module was replaced with a lightweight C3k2 module by reducing the size of the convolutional kernel or adjusting the branching structure. Second, a new C2PSA module was added after the SPPF module, which combines cross-stage connectivity and a pixel-level self-attention mechanism to enhance its multi-scale feature fusion capability and improve its detection accuracy for targets of different sizes. Finally, the head module’s design was borrowed from the YOLOv10 model, which adopts depth-wise separable convolution and a dynamic label assignment strategy for efficient inference without NMS (non-maximal suppression) while reducing the number of parameters to detect and output three small targets with scales of 80×80, medium targets with scales of 40×40, and large targets with scales of 20×20, respectively. Therefore, the YOLO11n network structure was chosen as the base model in this study.

### 2.2. FPFS-YOLO

**Figure 2:**
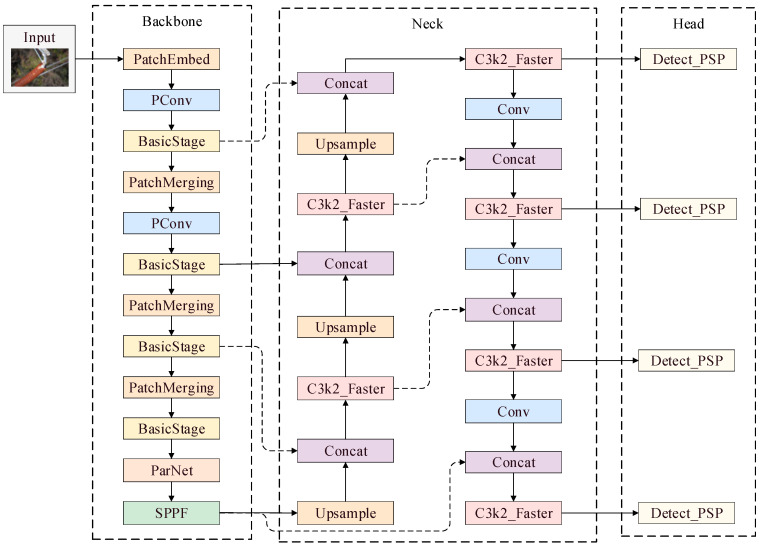
A diagram of FPFS-YOLO’s network structure.

To solve the problem of low mAP@50 values, precision, and recall due to leakages and the misdetection of defects in insulator images taken via UAV aerial photography, in this study, the FasterNet network, the ParNet attention mechanism, the small-defect detection head, and the lightweight C3k2_faster and PSP-Head modules are improved based on YOLO11n. The improved FPFS-YOLO network structure is shown in Figure 2.

### 2.3. Improvements with the FasterNet Network

**Figure 3:**
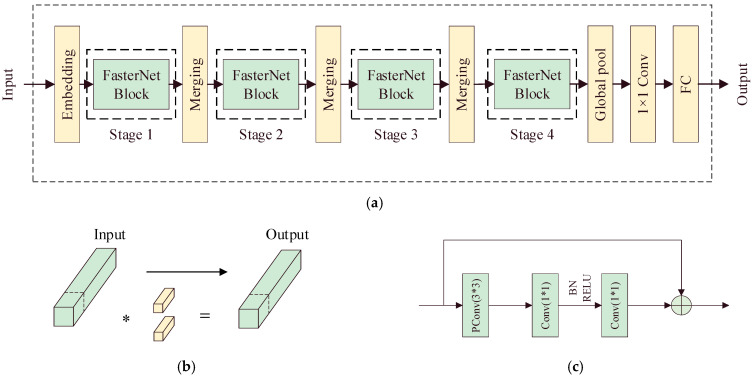
FasterNet’s overall architecture. (**a**) FasterNet network structure. (**b**) Diagram of the Partial Convolution (PConv) modules. (**c**) Diagram of the FasterNet block modules.

Aiming to address the problem of the backbone network of YOLO11n having a large computational volume and insufficient capability to extract insulator defect features, this study introduces the FasterNet lightweight network as an improvement strategy, so that the model can extract the features of insulator defects more efficiently while reducing the computational volume and the number of parameters. The overall architecture of FasterNet is shown in Figure 3. From Figure 3a, it can be seen that the FasterNet neural network is divided into four stages, each with a FasterNet block module and embedding or merging at its input, used for feature fusion and feature information organization at different levels. After the Stage 4 operation, the image features enter the last three layers: first, the global pooling layer (Global pool) for the aggregation of spatial information; then, the 1×1 Conv layer to adjust the number of channels; and finally, the fully connected layer for the feature map output. The FasterNet block consists of one PConv and two 1×1 Conv operations.

Compared to other lightweight networks such as MobileNets [23], ShuffleNets [24], etc., which use depthwise separable convolution (DWConv) or grouped convolution (GConv) to reduce FLOPS, FasterNet increases the amount of memory accessed and reduces the computational speed. The FasterNet neural network uses a PConv operation, which performs the regular Conv operation only on some of the channels in the feature map, leaving the rest unchanged, and finally fuses all of the channel information through PWConv. This effectively reduces the number of FLOPS and the amount of memory accessed.

PConv usually selects the first or the last consecutive channel as the representative feature map when accessing memory consecutively or periodically. It effectively reduces the computational complexity while maintaining feature representation, as it avoids the redundant computation of traditional convolution by selectively processing the spatial features of the input channels, making it more suitable for real-time target detection tasks. That is, PConv is able to extract spatial features more efficiently while reducing its computation and memory access; therefore, in this study, PConv is added to the backbone network.

The FLOPS ratios for PConv and Conv are shown in Equation (1):

(1) $$\frac{FLOPs_{\mathrm{PConv}}}{FLOPs_{\mathrm{Conv}}}=\frac{h\times w\times k^{2}\times c_{p}^{2}}{h\times w\times k^{2}\times c^{2}}$$

where h is the width of the feature map, w is the height of the feature map, k is the size of the convolution kernel, and $c_{p}$ is the number of channels in the convolution operation. When the proportion of channels involved in the convolution is $r=\frac{c}{c_{p}}=\frac{1}{4}$, PConv’s FLOPS value is $\frac{1}{16}$ of Conv’s.

The amount of memory accessed by PConv is shown in Equation (2):

(2) $$h\times w\times 2c_{p}+k^{2}\times c_{p}^{2}\approx h\times w\times 2c_{p}$$

When $r=\frac{1}{4}$, the amount of memory accessed by PConv is about $\frac{1}{4}$ of that for Conv.

The above formulas illustrate that PConv significantly reduces computational complexity while retaining information for channels not involved in the convolution so that the integrity of the features can be maintained.

Since the detection accuracy decreases after replacing the backbone with the FasterNet lightweight network, this study seeks to improve the FasterNet neural network. Firstly, PConv operations are added after the first two embedding steps because the embedding layer can map the input data to a low-dimensional feature space, which is an important part of feature extraction. We chose to add PConv after the first two embedding steps in this study to protect the integrity and avoid excessive loss of the initial features while also reducing redundant information. The addition of these two PConv operations to the FasterNet lightweight network improves the detection accuracy.

### 2.4. ParNet Attention Mechanism and Small-Defect Detection Head

#### 2.4.1. ParNet Attention Mechanism

**Figure 4:**
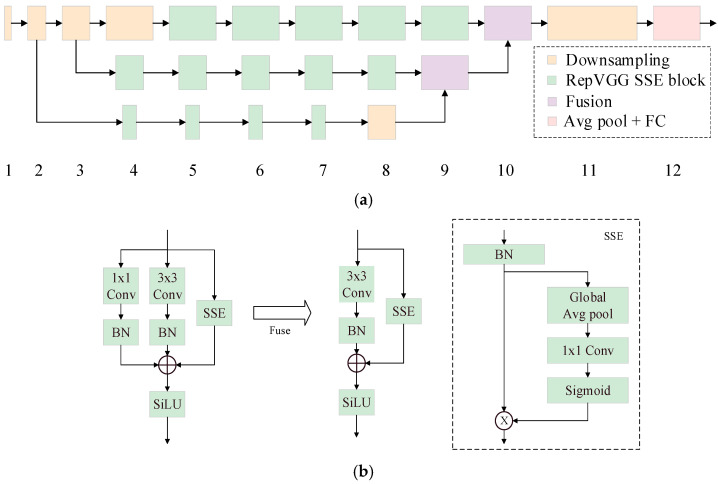
The ParNet (Parallel Attention Network) attention mechanism. (**a**) ParNet module diagram. (**b**) The ParNet block module diagram.

To address the insufficient sensory field and loss of detail when detecting small targets and to better model long-range dependencies, this study introduces the ParNet (Parallel Attention Network) attention mechanism proposed by Coyal et al. [25], the structure of which is shown in Figure 4.

As shown in Figure 4a, ParNet is a parallelized neural network architecture that employs three branches, each dealing with different input features, and avoids the gradient coupling problem caused by serial networks. As shown in Figure 4b, the ParNet module has a schematic structure. First, the ParNet module has two parallel paths: One starts with the 1×1 Conv layer and then the normalization layer (BN). The 1×1 Conv operation is able to efficiently extract global features, reduce the amount of computation and memory consumed, and optimize large-scale processing of images. The other path starts with the 3×3 Conv layer, followed by a normalization layer (BN), where the 3×3 Conv is able to capture the local spatial information from the feature maps, enabling the model to better understand the local structure of insulator defects. Subsequently, the processed results are fused into the results obtained after branching into the Spatial Squeeze Excitation (SSE) module, which is a step that fuses different scales and types of features to enhance insulator defect imaging. Finally, merging the output from the SiLU activation function enhances its nonlinear expression so that it can better capture the features in complex environments, thus improving the detection accuracy. After the fuse operation, the 1×1 Conv branch is removed, and only the 3×3 Conv and BN are retained before connecting the SSE module and the SiLU activation function, which effectively reduces the model’s computational cost and complexity, as well as saves memory.

The SSE module first normalizes (BN) the feature map, and then, the processed features are subjected to global average pooling (the Global Avg pool); this step compresses the feature map into 1×1, and then, 1×1 Conv initiates feature correlation extraction and generates weights through the Sigmoid activation function. This module enhances the feature representation for insulator defects, accurately captures insulator defects by assigning channel weights and optimizing the feature fusion, enhances the generalizability of the model, and adaptively adjusts to different types of environments and interferences in order to detect insulator defects in complex backgrounds.

In this study, the ParNet attention mechanism is added in front of the SPPF module, which combines multi-scale feature fusion and channel attention to effectively enhance the feature extraction ability of the model for insulator defects, which not only improves its detection accuracy but also enhances its ability to cope with complex backgrounds and different types of environmental disturbances and provides a more effective solution for insulator defect detection tasks.

#### 2.4.2. Small-Defect Detection Head

Due to the limitations of the YOLO11n model in feature extraction, it is easy to lose details, such as small defects, in an image. Thus, when aerial images captured during UAV operations contain small, differently shaped insulator defects with features that are not prominent and are difficult to capture, the model will falsely detect leakages due to small defects.

In this study, the initial image size input into the YOLO11n model was 640×640×3, and after 8, 16, and 32 instances of downsampling, feature map sizes of 80×80, 40×40, and 20×20 were acquired, respectively, corresponding to small, medium, and large targets in the head. A shallow detection head with 4 instances of downsampling was added to detect small defects on insulators, which increased the feature extraction capability of the model for small defects on insulators, and the corresponding feature map was 160×160 in size. Although the addition of a small-defect detection layer lead to an increase in the amount of computation and the number of parameters, the accuracy for insulator small defect detection was significantly improved.

### 2.5. Lightweight C3k2_Faster and PSP-Head Modules

#### 2.5.1. Lightweight C3k2_Faster Module

**Figure 5:**
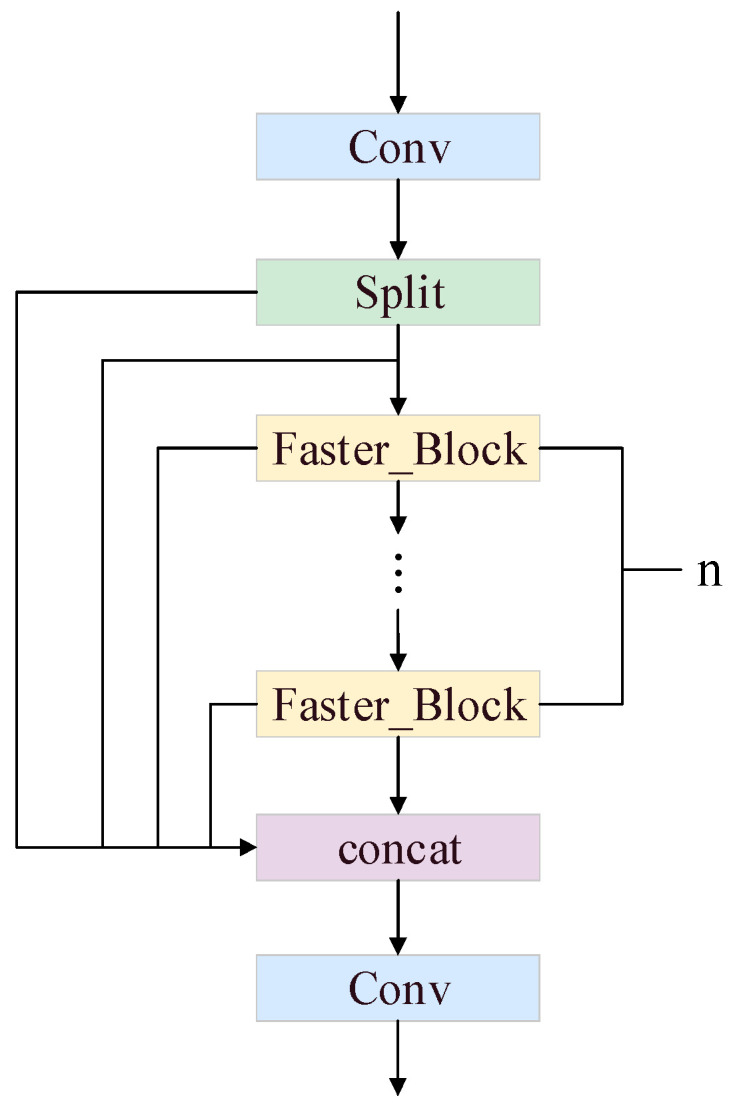
Structure of the C3k2_faster module.

Due to problems such as computational redundancy caused by the introduction of the ParNet attention mechanism and the small-defect detection layer into the model, in this study, based on the FasterNet lightweight network structure, we propose the C3k2_faster module, which inherits the structure of C3k2 but replaces C3k with Faster_Block, which consists of PConv and PWConv. This creates a more efficient feature processing flow in which PConv is responsible for extracting the spatial features, and PWConv then fuses the features across channels to maintain the feature expression capability at reduced computational costs. A diagram of the model’s structure is shown in Figure 5.

#### 2.5.2. Lightweight PSP-Head

**Figure 6:**
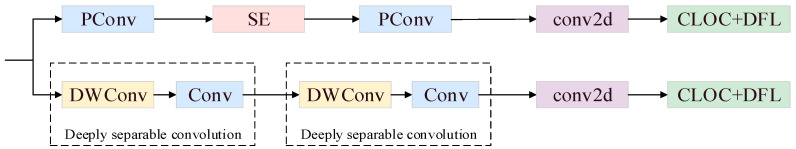
PSP-Head structural model.

The detection head of the YOLO11n model is processed through separate branches for classification and regression tasks, and such a structure can cause problems such as computational redundancy, conflicting feature calls, or inconsistency between accurate localization and misclassification, which ultimately affects the model’s detection ability, especially when two 3×3 Conv layers are used for extracting features during a regression task, leading to an increase in the amount of computation needed. Therefore, this study proposes an improved detection head design, PSP-Head, of which a structural model diagram is shown in Figure 6. PSP-Head simultaneously hones the detection accuracy of the model while reducing the computational burden and parameter count.

**Figure 7:**
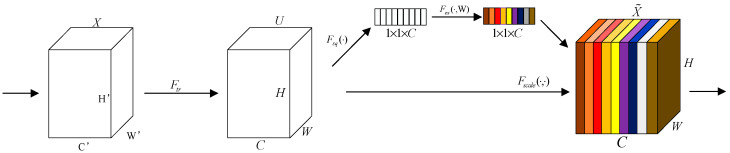
SE Attention Mechanism.

In this study, the introduction of PConv significantly reduces the computational cost of the model, while the introduction of the SE attention mechanism [26] enhances the model’s ability to extract features. The latter is shown in Figure 7. The input features first enter PConv, which are divided into a processing group and a retention group; the retention group retain the original feature information from the input, while some of the features in the processing group undergo convolution to downscale the amount of computation. After fusing these two groups of features into the SE attention mechanism, the module contains both a channel attention and spatial attention mechanism: the channel attention mechanism learns the correlations between channels through global average pooling and two fully connected layers, and the spatial attention mechanism captures the spatial importance through the fusion of maximum pooling and average pooling. The output features are fed through another PConv operation for feature extraction, and this step not only maintains computational efficiency but also accelerates the training convergence speed. As a result, the feature fusion improves significantly.

Using this method, not only are the number of parameters and calculations reduced, but also the detection accuracy is improved, which is especially important for applications in the real-time detection of insulator defects.

## 3. Experimental Design and Validation

### 3.1. Dataset

In this study, the insulator aerial photography data was selected from the China Power Transmission Line Insulator Dataset (CPLID), some of which were obtained during UAV inspections of insulator defects in the transmission lines. Insulator defects fall into five categories: pollution-flashover, broken, self-explosion, guano, and entanglement. Due to the limited scenarios covered by the training data, the generalization ability of the model will also be affected. The model may fail to accurately identify targets with low occurrence frequencies or those different from the training samples, thereby reducing the overall performance of the algorithm. In order for the model to better learn insulator defect features, this study uses spatial geometric transformation, adding noise, image aliasing, and adjusting the brightness and other operations to expand the data in the dataset from 832 images to 3236. Among them, the spatial geometric transformation is flipping and rotation, the noise addition is impulse noise, and the image aliasing is transformed by the Mosaic-9 data augmentation method, including operations such as contrast adjustment, scaling and cropping, and arranged according to the rules to obtain a new image with uniform size. Meanwhile, the dataset is divided into training, validation, and test sets at a ratio of 7:2:1.

### 3.2. Experimental Environment and Evaluation Indicators

The device configuration involved an NVIDIA GeForce RTX 4060 Ti GPU, a 12th Gen Intel(R) Core (TM) i9-12900F CPU, Windows 10, PyTorch framework version 2.0.0+cu118, Python version 3.10.17, and the experimental parameters shown in Table 1.

The following evaluation metrics are often used for target detection models: the mean average precision (mAP, mean average precision), precision, recall, parameter counts, and Floating Point Operations (GFLOPS).

In this study, mAP@50 is used as the key evaluation metric, with the mAP values calculated at an IoU threshold of 0.5.

### 3.3. Comparative Experiments

In order to evaluate the performance of the FPFS-YOLO model in insulator defect detection, this study compares it with commonly used models such as RT DETR, YOLOv3-tiny, YOLOv5n, YOLOv6n, YOLOv8n, YOLOv9t, YOLOv10n, YOLO11n, YOLO11s, and YOLO12n. In the table, each group of experiments used the same experimental environment and parameters. The experimental results are shown in Table 2.

As can be seen from Table 2, the FPFS-YOLO model proposed in this study achieves 2.2%, 13.32%, 30.9%, and 33.2% higher mAP@50 values compared to the RT DETR, Nanodet-m, Mobilenet-yolo, and DEIM models, respectively. Moreover, it demonstrates significantly higher computational resource requirements, indicating that these four models perform poorly in the detection of insulator defects. YOLOv5n, YOLOv9t, YOLOv10n, and YOLO12n have 0.31M, 0.84M, 0.12M, and 0.25M fewer parameters than the FPFS-YOLO model, respectively. YOLOv5n, YOLOv9t, and YOLOv10n cost 0.2G, 0.7G, and 1.3G more computationally than the FPFS-YOLO model, while YOLO12n cost 0.6G less computationally; however, mAP@50 was lower for all four models. At the same time, YOLOv3-tiny, YOLOv6n, and YOLOv8n incur more computational costs and require more parameters, and their mAP@50 values are lower, which makes them unsuitable for insulator defect detection. The mAP@50 value for the YOLO11s model is 91.5%, but its computational cost and parameter count are considerable. The FPFS-YOLO model showed a 3.1% improvement over the YOLO11n model in terms of mAP@50, a 1.2% improvement for mAP@0.5-0.95, a 1.5% improvement in precision, and a 4.2% improvement in recall, which demonstrates the effectiveness of our improvements.

Of the 10 models mentioned above, the FPFS-YOLO model has the highest mAP@50, at 91.5%, indicating the lowest false detection rate, the highest recall rate, and the lowest missed detection rate. Therefore, the FPFS-YOLO model achieves a balance between being accurate and lightweight, making it more suitable for the detection of insulator defects in complex scenarios.

### 3.4. Ablation Experiments

In order to verify the effectiveness of the FPFS-YOLO model, ablation experiments were carried out on the basis of the YOLO11n model to verify the influence of each module on performance. In the table, each group of experiments used the same experimental environment and parameters. The experimental results are shown in Table 3. The symbol “√” denotes that the corresponding module is enabled. The symbol “×” indicates that the corresponding module is not enabled.

Experiment 1 utilized the YOLO11n model without any modifications, Experiment 2 represented the model with the introduction of the FasterNet neural network, Experiment 5 used the improved FasterNet backbone network (from Experiment 4, combining FasterNet, PConv, and ParNet) with the small-defect detection head, Experiment 6 incorporated the PSP_Head, Experiment 7 added the C3k2_Faster, and Experiment 8 introduced the FPFS-YOLO model proposed in this study, which integrates both PSP_Head and C3k2_Faster.

As can be seen from Table 3, the mAP@50 values achieved in Experiments 3-8 were all higher than those in Experiment 1, and the mAP@50 value for Experiment 2 was lower than that achieved in Experiment 1; however, the parameter count and computational cost were lower than those in Experiment 1, showing that each of the improved modules can be effective in the detection of defects in insulators.

First, when the FasterNet lightweight network was introduced, mAP@50 decreased by 0.5% but the parameter count of the model decreased by 0.25M and the computational cost decreased by 0.3G, indicating that while the FasterNet lightweight network reduced the computational cost, it also limited the model’s ability to extract features. To further optimize the model and improve its accuracy, the PConv module was subsequently introduced. PConv increased the mAP@50 by 0.2% but reduced its computational cost and parameter count by 2.7G and 0.64M, respectively. The PConv module can improve the computational efficiency while maintaining the mAP@50 value. To address issues such as an insufficient sensing field and a loss of detail in insulator defect detection, the ParNet attention mechanism was introduced in this paper. The mAP@50 value, the parameter count, and the computational cost increased by 0.9%, 0.72M, and 0.6G, respectively, demonstrating the effectiveness of this introduction. Additionally, the introduction of the small-defect detection head boosted mAP@50 by 1.0%, albeit with increases of 0.08M in parameters and 3.8G in computational load. While mAP@50 showed improvement, the computational cost still required further optimization. The computational cost still needed to be further reduced. Thus, when C3k2_Faster and PSP_Head were introduced in this study, the best balance in the performance of the model was achieved, with a 3.1% improvement in mAP@50 while retaining a computational load of 7.2G and a parameter size of 2.81M. To sum up, the improved FPFS-YOLO model achieved a significant improvement in performance, enabling more accurate detection of insulator defects and precise determination of their categories, thereby more effectively addressing the challenges of practical applications.

### 3.5. Visualization of the Results

**Figure 8:**
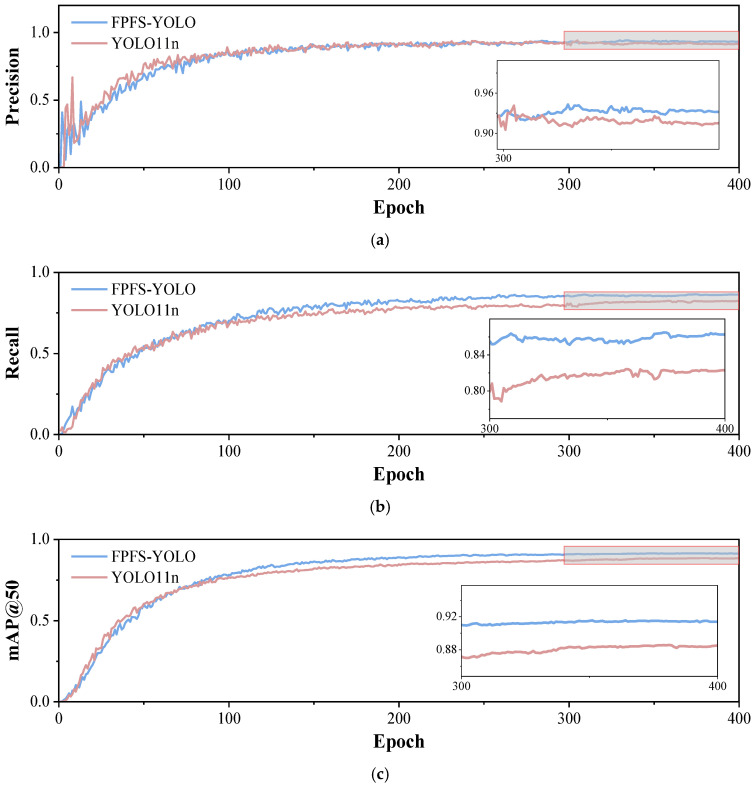

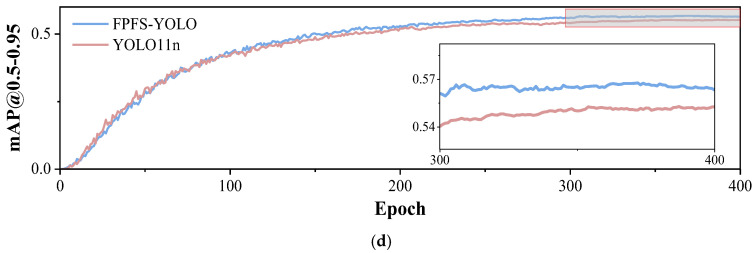
Comparison of evaluation metrics for YOLO11n and FPFS-YOLO. (**a**) Precision comparison chart. (**b**) Recall comparison chart. (**c**) mAP@50 comparison chart. (**d**) mAP@0.5-0.95 comparison chart.

The variation in performance for the FPFS-YOLO and YOLO11n models across training rounds based on different evaluation metrics is shown in Figure 8.

A comparison of the precision results is shown in Figure 8a. After 300 rounds of training, the accuracy of the FPFS-YOLO model begins to surpass that of the YOLO11n model and remains higher throughout subsequent training. A comparison of the recall results is shown in Figure 8b. The FPFS-YOLO model shows a slightly higher recall rate throughout the training process, indicating that it is slightly superior in identifying positive samples. Eventually, the recall rates for both models converge to 0.8 at the end of the training, demonstrating improved recognition. In the comparison of mAP@50 values in Figure 8c, when the number of training rounds approached 100, the mAP@50 value of the FPFS-YOLO model surpassed that of the YOLO11 model, at 0.915, while the training result for the YOLO11n model was 0.884. The comparison of mAP@0.5-0.95 values in Figure 8d indicates that the mAP@50 value for the FPFS-YOLO model was slightly higher than that for the YOLO11n model throughout the training process, especially after 100 rounds of training, and ultimately, the mAP@0.5-0.95 values of both models were close to 0.6. To sum up, on the whole, the FPFS-YOLO model not only maintains a higher recall rate than the YOLO11n model but also boasts significantly improved detection accuracy and classification accuracy. This indicates that the FPFS-YOLO model can detect insulator defects better.

### 3.6. Comparison of the Detection Effect

**Figure 9:**
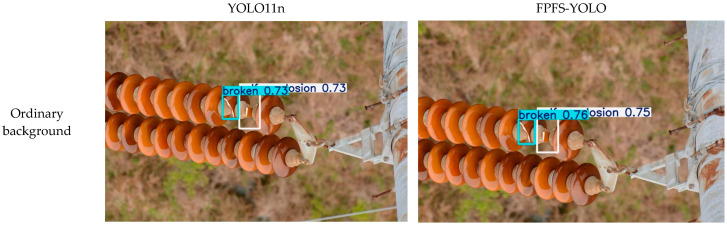

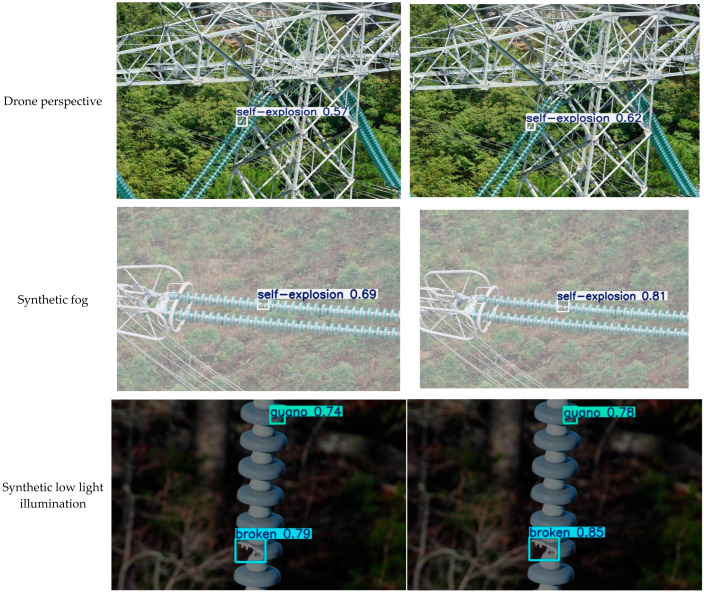
Detection effects of YOLO11n and FPFS-YOLO against different backgrounds.

In order to verify the detection effect of the FPFS-YOLO model against different backgrounds, images requiring more complex detection were selected to validate the model in this study. The results are shown in Figure 9.

Under normal background conditions, the confidence level of the FPFS-YOLO model is slightly higher than that of the YOLO11n model. In the detection of damaged and self-bursting insulators, the confidence level of the YOLO11n model is 0.73 for both cases, while that of the FPFS-YOLO model is 0.76 and 0.75, respectively, increasing by 0.3% and 0.2%. This indicates that the FPFS-YOLO model has higher accuracy. From the perspective of the unmanned aerial vehicle, the confidence level of the FPFS-YOLO model in detecting self-explosion insulators is 0.62, which is 5% higher than that of the YOLO11n model. This indicates that the FPFS-YOLO model can provide more reliable detection results for subsequent processing. In the images of synthetic fog, the confidence of the FPFS-YOLO model in identifying the blown-off insulator was 0.81, while that of the YOLO11 model was 0.69, an increase of 12%. In the images of synthetic low light, the confidence of the FPFS-YOLO model in detecting guano and broken parts was 0.78 and 0.85, respectively, which were 4% and 6% higher than that of the YOLO11 model. This indicates that the FPFS-YOLO model is robust and can provide more reliable detection results for subsequent processing.

The visualization results show that the FPFS-YOLO model outperformed the YOLO11n model in terms of its detection accuracy, robustness, and generalization ability, which enables it to provide more stable and accurate results in practical applications and to better adapt to scenarios involving insulator defect detection using UAVs.

## 4. Discussion

This study proposed an FPFS-YOLO model based on the YOLO11n model. Aiming to address the problem of the low detection accuracy of the YOLO11n model, the FasterNet lightweight network, PConv, the ParNet attention mechanism, and a small-defect detection layer were added to the network structure. The model’s ability to extract and fuse small-defect features was enhanced through the multi-level network, as was its accuracy in detecting small defects. Aiming to solve problems such as computational redundancy caused by introducing the ParNet attention mechanism and the small-defect detection layer into the model, the C3k2_faster module was introduced, and the PSP-Head detection head was designed, which further improved the detection accuracy while reducing the model’s computational cost and parameter count. The results show that the FPFS-YOLO model achieved a 91.5% mAP@50, which is 3.1% higher than that achievable using the YOLO11n model. Meanwhile, the precision and recall were improved by 1.5% and 4.2%, respectively, but the model’s parameter count reached 2.81 M, an increase of 0.23 M. Compared with the YOLO11n model, the FPFS-YOLO model improves the accuracy, recall rate and precision rate, but the number of parameters still needs to be further reduced. Future work could optimize the performance of the model under extreme-weather or low-light conditions and further reduce the number of parameters of the model. Deploying the model to unmanned aerial vehicles (UAVs) can enable it to be widely applied in practical scenarios such as power inspection and UAV inspection, thereby achieving more efficient detection of insulator defects.

## Figures and Tables

**Table 1:** Experimental parameter settings.

Parameters	Setpoint
Training rounds	400
Batch size	8
Image size	640×640
Initial learning rate	0.01
Optimizers	SGD
Momentum	0.937
Weight Decay	0.0005
Warmup Epochs	3.0
Confidence Threshold	0.25
Close_mosaic	10

**Table 2:** Performance comparison of various models for insulator defect detection.

Model	Param/M	GFLOPs/G	P/%	R/%	mAP@50/%	mAP@0.5-0.95/%
RT DETR	41.94	125.6	90.8	84.1	89.3	56.3
Nanodet-m	0.93	1.38	64.01	70.52	78.18	33.64
Mobilenet-yolo	11.33	8.47	89.79	24.68	60.6	22.5
DEIM	3.7	7.12	80.6	38.2	58.3	24.1
YOLOv3-tiny	12.13	18.9	91.6	77.1	82.6	51.6
YOLOv5n	2.5	7.1	91.1	82.8	87.9	54.9
YOLOv6n	4.23	11.8	91.9	79	84	52.3
YOLOv8n	3.01	8.1	94.6	83.1	89.2	56.2
YOLOv9t	1.97	7.6	91.4	84.1	88.5	56.1
YOLOv10n	2.69	8.2	93.3	82	88	56.1
YOLO11n	2.58	6.3	91.7	82.2	88.4	55.4
YOLO11s	9.41	21.3	94.3	86.6	91.5	59.1
YOLO12n	2.56	6.3	91.9	81.7	86.9	54.7
FPFS-YOLO	2.81	6.9	93.2	86.4	91.5	56.6

**Table 3:** A comparison of the ablation experiment results for the methods in this study.

Experiment	Faste rNet	PConv	ParNet	Small-Defect Detection Head	C3k2_Faster	PSP_Head	Param/M	GFLOPs/G	mAP@50/%
1	×	×	×	×	×	×	2.58	6.3	88.4
2	√	×	×	×	×	×	2.33	6	87.9
3	√	√	×	×	×	×	2.34	6.1	89.1
4	√	√	√	×	×	×	3.06	6.7	90.0
5	√	√	√	√	×	×	3.14	10.5	91.0
6	√	√	√	√	×	√	2.87	7.2	90.7
7	√	√	√	√	√	×	3.24	11.5	91.0
8	√	√	√	√	√	√	2.81	6.9	91.5

## Data Availability

The data are included in this article.
